# The health benefits of secondary education in adolescents and young adults: An international analysis in 186 low-, middle- and high-income countries from 1990 to 2013

**DOI:** 10.1016/j.ssmph.2016.12.004

**Published:** 2016-12-14

**Authors:** Russell M. Viner, Dougal S. Hargreaves, Joseph Ward, Chris Bonell, Ali H. Mokdad, George Patton

**Affiliations:** a UCL Institute of Child Health, 30 Guilford St., London WC1N 1EH, UK; b London School of Hygiene and Tropical Medicine, Keppel St, London WC1E 7HT, UK; c Institute of Health Metrics & Evaluation, 2301 Fifth Ave., Suite 600, Seattle, WA 98121, USA; d Centre for Adolescent Health, Royal Children's Hospital, 50 Flemington Rd, Parkville, Vic 3052, Australia

**Keywords:** Global, Adolescent, Secondary education, Adolescent fertility, Mortality, HIV

## Abstract

**Background:**

The health benefits of secondary education have been little studied. We undertook country-level longitudinal analyses of the impact of lengthening secondary education on health outcomes amongst 15-24 year olds.

**Methods:**

Exposures: average length of secondary and primary education from 1980 to 2013.

Data/Outcomes: Country level adolescent fertility rate (AFR), HIV prevalence and mortality rate from 1989/90 to 2013 across 186 low-, middle- and high-income countries.

Analysis: Longitudinal mixed effects models, entering secondary and primary education together, adjusted for time varying GDP and country income status. Longitudinal structural marginal models using inverse probability weighting (IPW) to take account of time varying confounding by primary education and GDP. Counterfactual scenarios of no change in secondary education since 1980/1990 were estimated from model coefficients for each outcome.

**Findings:**

Each additional year of secondary education decreased AFR by 8.4% in mixed effects models and 14.6% in IPW models independent of primary education and GDP. Counterfactual analyses showed the proportion of the reduction in adolescent fertility rate over the study period independently attributable to secondary education was 28% in low income countries. Each additional year of secondary education reduced mortality by 16.9% for 15-19 year and 14.8% for 20-24 year old young women and 11.4% for 15-19 year and 8.8% for 20-24 year old young men. Counterfactual scenarios suggested 12% and 23% of the mortality reduction for 15-19 and 20-24 year old young men was attributable to secondary education in low income countries. Each additional year of secondary education was associated with a 24.5% and 43.1% reduction in HIV prevalence amongst young men and women.

**Interpretation:**

The health benefits associated with secondary education were greater than those of primary education and were greatest amongst young women and those from low income countries. Secondary education has the potential to be a social vaccine across many outcomes in low and middle income countries.

## Highlights

• Greater primary education is associated with health benefits.
• Studies suggest that education may have causal effects on health.
• The health benefits of secondary education are greater than for primary education.

## 1 Introduction

Education is suggested to be one of the strongest determinants of health and human capital (Commission on Social Determinants of Health, 2008), with the association of better education with greater health and wellbeing seen across the life-course and across very different socioeconomic, cultural and political contexts and one that has persisted over time (Montez & Friedman, 2015). The more educated live longer lives with less disability and ill-health in both rich and poor countries, and there is evidence that the association between education and health is strengthening over time in high income countries (Strand, Groholt, Steingrimsdottir, Blakely, Graff-Iversen & Naess, 2010). Education appears to also have inter-generational benefits; improved education for women may account for up to half the global improvement in child mortality since 1970 (Gakidou, Cowling, Lozano, & Murray, 2010). For this reason universal primary education was one of the key UN Millennium Development Goals (MDG Goal 2).

There is a growing consensus that education has some causal effects on health, (Baker et al., 2011, Behrman, 2015, Miyamoto and Chevalier, 2010, Montez and Friedman, 2015) although there are also likely pathways from health to education and confounding by genetic (Liu et al., 2015) or personality factors (Fuchs, 1982) contributing to both improved health and higher educational attainment. Education may improve health through multiple mechanisms, including stimulation of greater cognitive development and self-regulation, knowledge acquisition and literacy, promotion of more healthy behaviours and avoidance of health risks, greater access to protective societal resources (e.g. the built environment or medical care), avoidance of early marriage as well as greater exposure to prosocial peers and enhancement of social support networks (Baker et al., 2011; Jukes, Simmons, & Bundy, 2008; Liu et al., 2015; Miyamoto & Chevalier, 2010). Through these or alternative mechanisms, education may modify genetic risks for certain diseases (Liu et al., 2015). Education may also be most powerful when substituting or compensating for deprived backgrounds (Ross & Mirowsky, 2011). The existence and strength of such mechanisms are almost certainly dependent on broader social and economic contexts, and are likely to vary by national development level and income (Montez & Friedman, 2015; Smith-Greenaway, 2015).

Many of the suggested mechanisms by which education may influence health can be argued to be most strongly operative during secondary schooling, given dramatic cognitive development during adolescence and the emergence of key health issues such as substance use, depression, sexually transmitted infections and teenage pregnancy emerge during this time (Patton et al., 2016). Yet health gains from secondary education have been little studied, in contrast to primary education. This is despite a dramatic global expansion in length of education in the past 30 years, with most gains in the late primary and early secondary years (Institute for Health Metrics and Evaluation (IHME), 2015). Data from the Institute of Health Metrics (IHME) suggests that globally in 2015, young women aged 15-24 years had on average 9.5 years of education and young men had 9.9 years. Less than 7 average years of education, equivalent to primary education, was the norm for young men in 22% of countries and for young women in 26% of countries. Achieving 8-10 years of education, equivalent to lower secondary, was the norm for 34% of countries for young men and 18% for young women, with upper secondary or beyond (11 plus years of education) being the norm in 44% of countries for young men and 56% for young women (Institute for Health Metrics and Evaluation (IHME), 2015). Amongst adults in high income countries (HIC) upper secondary education is the education level most strongly associated with better health and mental health, (Miyamoto & Chevalier, 2010) although tertiary education confers additional benefits in US studies (Case & Deaton, 2015). Secondary education is known to promote better pregnancy and child health outcomes amongst adult women internationally, (Grepin and Bharadwaj, 2015, UNESCO, 2010) and a small literature from Sub-Saharan African countries suggests that the effect of secondary schooling on teenage fertility may be stronger and more consistent than for primary education (Mahy & Gupta, 2002). However the effects of education on health may differ across cultures and nations, (Snopkowski, Towner, Shenk, & Colleran, 2016) and may be greater in resource poor groups and settings (Ross & Mirowsky, 2011).

Estimates that poor education attainment is a relatively large contributor to overall health, e.g. directly contributing to 9% of US deaths in a recent study, (Galea, Tracy, Hoggatt, Dimaggio, & Karpati, 2011) have driven interest in education policy as a potential tool to improve population health (Miyamoto & Chevalier, 2010; Montez & Friedman, 2015). The United Nations Sustainable Development Goals (SDG) include a target for countries to provide every child with access to free primary and secondary education by 2030 (Target 4), (Sustainable Development Knowledge Platform, 2014) driven by the well-evidenced benefits of education for economic development (Barro, 2013). Concerns about the ability of low income countries to meet this target led to the formation of the International Commission on Financing Global Education in 2015 (Brown, 2015).

Here we undertake the first systematic international exploration of the effect of participation in secondary education on health outcomes in adolescence and young adulthood. We used country-level analyses to examine longitudinal associations over the past 23 years of average length of secondary compared with primary education per country with key SDG priority outcomes, adolescent fertility rate, HIV prevalence and all-cause mortality, amongst 15-24 year olds. We undertook separate analyses by gender given differences in length of education for women, (Institute for Health Metrics and Evaluation (IHME), 2015) differences in causes of mortality between sexes in adolescence, (Viner et al., 2011) and previously reported differences in education and health associations (Ross, Masters, & Hummer, 2012). We undertook longitudinal analyses and examined trends by world region and by country income status as associations between health and education may shift with economic development (Snopkowski et al., 2016). We hypothesized that greater length of participation in secondary education in a country was associated with better health outcomes for a country over and above benefits from primary education and after accounting for economic development. We tested these hypotheses in a range of models for each health outcome studied, and used data from the models to compute counterfactual estimates for each health outcome, identifying the likely contribution of increases in secondary education to progress in each health outcome by world region and by country income group.

## 2 Methods

We used longitudinal models to examine associations between secondary education and health outcomes globally.

### 2.1 Education data

Recent and reliable national education data on a wide range of countries were available from two sources:

I. Secondary education: Barro and Lee (BL) (Barro & Lee, 2010) data on national average completed years in primary, secondary and tertiary education for 146 countries at 5 year intervals from 1950 to 2010. We interpolated education outcomes for intervening years to provide estimates for each country-year for 15-24 year olds (see Appendix).
II. Overall education: Estimates of the national average completed years of education for people over the age of 15 in 10 year age-bands from 1970 to 2015 were recently published by the Institute of Health Metrics and Evaluation (IHME) for 188 countries by sex (Institute for Health Metrics and Evaluation (IHME), 2015). For these analyses we used 15-24 year olds (Appendix).

Our primary analyses in this paper related to the secondary education data from the BL dataset, however we also included analyses using overall years of education (IHME) as most variation between countries in overall years of education now lies in the secondary education domain.

### 2.2 Health outcomes

Adolescent fertility rate (AFR), defined as annual births per 1000 women aged 15-19 years were obtained from the United Nations Department of Economic and Social Affairs Population Division for 1990 to 2012 (United Nations, 2014). AFR data were available for 137 countries for secondary education analyses.

Data on HIV prevalence for 15 to 24 year olds were obtained for 1990 to 2014 from World Bank Open Data (World Bank, 2015b). HIV data were available for 93 countries for secondary education analyses.

All-cause mortality data were taken from two sources. First, from age and sex specific all-cause mortality data from the Global Burden of Disease Study 2013 (GBD 2013). Mortality rates per 100,000 person years of observation were estimated from a variety of sources, including surveys, censuses, sample registration systems, disease surveillance, and vital registration systems (Mortality & Causes of Death, 2015) (see Appendix).

Secondly, given that GBD2013 mortality data were derived using modelling techniques that included education as a covariate (see Appendix), we conducted confirmatory analyses using unmodelled mortality data from national registration systems collected in the World Health Organisation (WHO) Mortality Database ("WHO Mortality Database," 2015). We included only country years with >70% completeness of vital registration, which limited this dataset to largely middle and high-income countries. For these analyses, data were available for 87 largely high and middle-income countries, with variable amounts of data available per country between 1980 and 2013 (in total 429 country-years: see Appendix).

### 2.3 Macroeconomic data

We adjusted analyses for macroeconomic factors likely to confound associations between education and health. These included gross domestic product (GDP) per capita as a measure of national wealth, and also country classification by income group (high, upper- or lower- middle and low income) in 1990; the latter was included as it was significant in models including GDP and likely provided additional adjustment for national development. We further adjusted models for national spending on health as a proportion of GDP, however this was undertaken in sensitivity analyses only as data were limited to 1995 to 2013 and only available for a subset of countries. All data were obtained from World Bank Open data from 1980 to 2014 (World Bank, 2015a) (see Appendix).

### 2.4 Analyses

Our primary analysis was of the effects of secondary compared with primary education on health outcomes, with secondary analyses of overall length of education. We first examined cross-sectional associations between education and health outcomes in 2010. Health outcomes were modelled as natural logarithms and models were adjusted for GDP and country income status.

Longitudinal associations were studied using multilevel models for change; such models use all available data from each country, allowing for uneven number of years available by country (Singer & Willett, 2003). First we ran models with BL data on secondary and primary education entered together. We followed (Gakidou, Cowling, Lozano & Murray, 2010) in including a random effect for country to account for determinants of young people’s health within each country, a random effect for time to account for technological progress and other time-varying factors that affect all countries and adjusting models for national income (GDP) as a major determinant of health outcomes that is unlikely to be on the causal pathway between education and health. GDP was modelled as mean GDP per capita for the previous 10 years in order to capture longer term effects of economic development. We also adjusted for country population and included country income status as a fixed effect as the impact of economic growth and growth in education on health may differ by baseline income status and inclusion improved model fit. Models were then repeated using data on overall years in education.

Second, in sensitivity analyses, we repeated these models including national health spending as a covariate however these had a low sample size. In mortality models, we also tested the effect of adding HIV prevalence, entered as a 3-year lagged prevalence in order to best capture effects of HIV on mortality (Gakidou et al., 2010).

Third, we repeated all analyses using inverse probability weighting (IPW) in structural marginal models to estimate the controlled direct effects of secondary education on health outcomes. The use of IPW constructs a pseudopopulation in which the exposure is independent of the factors included in the construction of the weighting, including time-varying confounders (GDP, primary education and population). The weighted regression models in the pseudopopulation can then be used to estimate the average causal effect of exposure in the original study population (Cole & Hernan, 2008).

Finally, we used the mixed effects model coefficients to compute counterfactual estimates for each health outcome relating to progress in secondary education, following the methods of (Gakidou et al., 2010). Counterfactuals were estimated by replacing the value of the average years of secondary education in all country years with the average years of secondary schooling in 1980 (for mortality) or 1990 (teenage fertility and HIV) for that country. Essentially we estimated progress over time in health outcomes if the education of young people had stayed constant at 1980/1990 levels. Counterfactual analyses were repeated by country income group and by UN region. Analyses were undertaken in Stata 14 (StataCorp, College Station TX).

## 3 Results

From 1980 to 2013, median years of overall education across all countries increased from 6.2 to 10.3 years amongst young men and from 5.9 to 10.9 years amongst young women 15-24 years of age (Appendix Figs. A1 & 2). During this time, secondary education increased from 2.0 to 4.1 years in young women and from 1.5 to 2.9 years amongst young men.

**Fig. 1:**
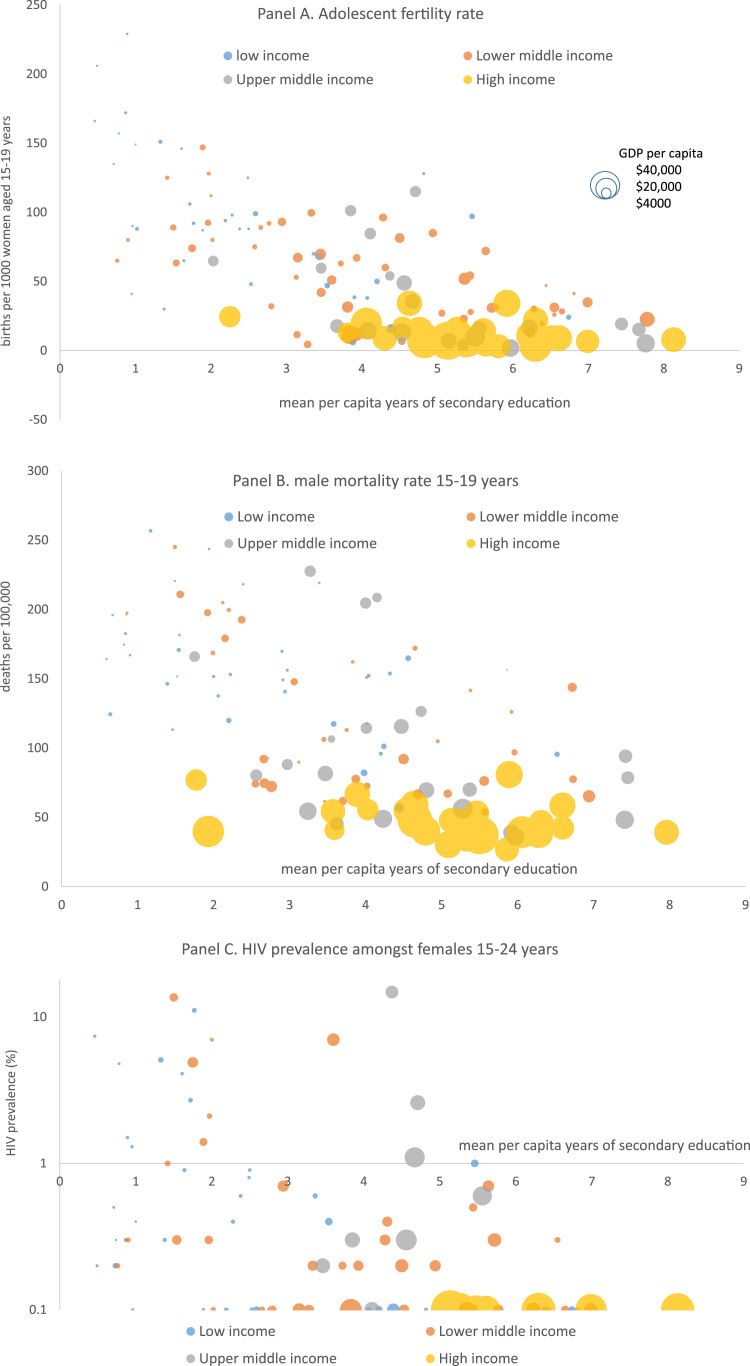
Health outcomes and years of secondary education per capita, by GDP and country income status.

Table 1 shows the cross-sectional associations of years of secondary and primary education and of overall years of education with each health outcome in 2010, adjusted for GDP and country income status. These associations are illustrated graphically for secondary education in Fig. 1 for adolescent fertility, mortality amongst 15-19 year old males and HIV prevalence in females (see Appendix Fig A3-9 for other outcomes). After adjusting for primary education, each additional year of secondary education was associated with a 16.8% reduction in adolescent fertility rate, 5.9-7.4% reduction in male mortality, 11.7-15.2% reduction in female mortality and 15.7% and 25.0% reductions in male and female HIV prevalence respectively. In each of these models, years of primary education was not significantly associated with adolescent fertility or mortality in either sex or with male HIV prevalence. Findings from analyses using WHO mortality data were similar (Appendix Table A1).

**Table 1:** Cross-sectional associations between average years of secondary and primary education per country and adolescent fertility rate, mortality and HIV prevalence in 2010 in males and females 15 to 24 years globally.

Outcome		N	Coefficient	Exp* coefficient	p		N	Coefficient	Exp* coefficient	p
**Adolescent fertility rate**	Primary years	137	−0.011	0.989	0.8	Years	178	−0.074	0.929	0.009
(log births/1000)	Secondary years	137	−0.184	0.832	<0.0001					
**Mortality rate**		140					182			
(log deaths/100,000)										
15-19 year males	Primary years		−0.023	0.978	0.4	Years		−0.055	0.947	<0.0001
	Secondary years		−0.077	0.926	<0.0001					
15-19 year females	Primary years		−0.036	0.964	0.2	Years		−0.086	0.918	<0.0001
	Secondary years		−0.125	0.883	<0.0001					
20-24 year males	Primary years		0.016	1.017	0.6	Years		−0.05	0.951	0.005
	Secondary years		−0.061	0.941	0.03					
20-24 year females	Primary years		0.010	1.010	0.8	Years		−0.099	0.905	<0.0001
	Secondary years		−0.165	0.848	<0.0001					
HIV prevalence (log %)		91					111			
Males 15-24 years	Primary years		0.314	1.369	0.05	Years		−0.094	0.91	0.15
	Secondary years		−0.171	0.843	0.003					
Females 15-24 years	Primary years		0.322	1.380	0.002	Years		−0.046	0.955	0.5
	Secondary years		−0.288	0.750	0.002					

Table Notes

Models included secondary and primary years of education entered together in the same model. All models adjusted for log of mean GDP for previous decade (2001-2010) and income status for each country.

Exp*: exponentiated coefficient

Longitudinal country-level models for the effects of secondary education on health outcomes are shown in Table 2 with models for overall education in Table 3.

**Table 2:** Longitudinal mixed-effect and structural marginal models for secondary years of education as predictors of adolescent fertility rate, mortality and HIV prevalence in 15 to 24 year olds.

		**Adolescent fertility rate**			**Male mortality**						**Female mortality**						**HIV prevalence**					
		log births per 1000 women 15-19 years			log deaths per 100,000 pyo						log deaths per 100,000 pyo						% population aged 15-24 years					
**Mixed effect models**																						
Country N			137		141						141						93					
Country-years			1702								4456						2133					
					4456																	
					15-19 years			20-24 years			15-19 years			20-24 years			males			females		
		*B*	*ExpB*	*p*	*B*	*ExpB*	*p*	*B*	*ExpB*	*p*	*B*	*ExpB*	*p*	*B*	*ExpB*	*p*	*B*	*ExpB*	*p*	*B*	*ExpB*	*p*
Constant		3.375			5.429			5.931			5.651			6.374			-3.240			-2.928		
Time (years)	linear	−0.014	0.986	0.02	0.000	1.000	0.9	0.002	1.002	0.5	−0.011	0.989	<0.001	−0.001	0.999	0.8	0.092	1.097	<0.001	0.006	1.006	0.5
	quadratic																−0.004	0.996	<0.001	−0.003	0.997	<0.001
*Education*																						
Primary education per capita (years)		0.027	1.027	0.3	−0.011	0.989	0.4	0.004	1.004	0.9	0.003	1.003	0.9	0.009	1.009	0.7	0.024	1.024	0.8	0.335	1.398	0.08
Secondary education per capita (years)		−0.088	0.916	0.002	−0.026	0.974	0.03	−0.039	0.962	0.003	−0.047	0.954	0.001	−0.050	0.951	0.02	−0.281	0.755	<0.001	−0.563	0.569	<0.001
GDP (log $ per annum)		0.184	1.202	0.006	−0.033	0.968	0.04	−0.053	0.948	0.05	−0.088	0.916	<0.001	−0.141	0.869	<0.001	0.253	1.288	0.07	0.282	1.326	0.05
Country status	Low income	0			0			0			0			0			–			–		
	Lower middle	−0.539	0.583	0.003	−0.255	0.775	0.001	−0.192	0.825	0.08	−0.593	0.553	<0.001	−0.650	0.522	<0.001	–			–		
	Upper middle	−1.194	0.303	<0.001	−0.139	0.871	0.3	−0.037	0.963	0.9	−0.702	0.495	<0.001	−0.773	0.462	<0.001	–			–		
	High income	−2.203	0.110	<0.001	−0.255	0.621	<0.001	−0.415	0.661	0.007	−1.138	0.321	<0.001	−1.240	0.289	<0.001	–			–		
Interaction of income with time		−0.005		0.04	−0.005		<0.001	−0.005		<0.001			0.2	−0.003		0.06	–			–		
**Inverse probability weighted (IPW) models**																						
		*B*	*ExpB*	*p*	*B*	*ExpB*	*p*	*B*	*ExpB*	*p*	*B*	*ExpB*	*p*	*B*	*ExpB*	*p*						
Secondary education		−0.158	0.854	<0.001	−0.121	0.886	<0.001	−0.092	0.912	<0.001	−0.185	0.831	<0.001	−0.160	0.852	<0.001						

Table notes

IPW models: models weighted for time-varying variables (log of lagged GDP, log of country population in each year, proportion not in education by country per year) and country income status, separately by sex.

IPW models were not estimated for HIV due to inclusion of a significant quadratic term in the models.

Income status not significant in mixed effects HIV models in either sex thus not included.

Pyo: person years of observation.

B: coefficient.

ExpB: exponentiated coefficient.

**Table 3:** Longitudinal mixed-effect and structural marginal models for overall years of education as predictors of adolescent fertility rate, mortality and HIV prevalence in 15 to 24 year olds.

	**Adolescent fertility rate**				**Male mortality**						**Female mortality**						**HIV prevalence**					
	log births per 1000 women 15-19 years				log deaths per 100,000 pyo						log deaths per 100,000 pyo						% population aged 15-24 years					
**Mixed effect models**																						
Country N			176		179						179						114					
Country-years			2031								5590						2621					
					5590																	
					15-19 year			20-24 years			15-19 years			20-24 years			15-24 years			15-24 years		
		*B*	*ExpB*	*p*	*B*	*ExpB*	*p*	*B*	*ExpB*	*p*	*B*	*ExpB*	*p*	*B*	*ExpB*	*p*	*B*	*ExpB*	*p*	*B*	*ExpB*	*p*
Constant		5.082			5.082			5.522			4.940			5.314			-3.483			-3.082		
Time (years)	linear	−0.015	0.985	<0.001	−0.001	0.999	0.7	0.005	1.005	0.17	−0.006	0.994	0.02	0.004	1.004	0.3	0.109	1.115	<0.001	0.120	1.127	<0.001
	quadratic																−0.004	0.996	<0.001	−0.004	0.996	<0.001
Education per capita (years)		−0.099	0.906	<0.001	−0.096	0.908	0.001	−0.120	0.887	<0.001	−0.057	0.945	0.008	−0.091	0.913	0.01	−0.245	0.783	<0.001	−0.246	0.782	<0.001
GDP (log $ per annum)		0.185	1.204	<0.001	−0.044	0.957	0.001	−0.058	0.944	<0.001	−0.093	0.911	0.004	−0.146	0.864	0.006	0.282	1.326	<0.001	0.220	1.246	0.05
Country income status	Low income	o			0			0			0			0			–			–		
	Lower middle	−0.381	0.683	0.01	−0.035	0.965	0.7	0.089	1.093	0.4	−0.533	0.587	<0.001	−0.457	0.633	0.002	–			–		
	Upper middle	−0.992	0.371	<0.001	0.004	1.004	0.9	0.108	1.115	0.4	−0.729	0.482	<0.001	−0.643	0.526	0.001	–			–		
	High income	−1.902	0.149	<0.001	−0.104	0.901	0.5	−0.157	0.854	0.4	−0.949	0.387	<0.001	−0.802	0.449	0.004	–			–		
Interaction of income status with time		−0.004		0.04																		
Inverse probability weighted (IPW) models *																						
		*B*	*ExpB*	*p*	*B*	*ExpB*	*p*	*B*	*ExpB*	*p*	*B*	*ExpB*	*p*	*B*	*ExpB*	*p*						
Education per capita (years)			−0.164	0.849	<0.001	−0.127	0.880	<0.001	−0.125	0.882	<0.001	−0.164	0.849	<0.001	−0.187	0.830	<0.001					

Table notes

IPW models weighted for time-varying variables (log of lagged GDP and log of country population in each year) and country income status, separately by sex.

IPW models were not estimated for HIV due to inclusion of a significant quadratic term in the models.

Income status not significant in mixed effects HIV models in either sex thus not included.

Pyo: person years of observation.

B: coefficient.

ExpB: exponentiated coefficient.

### 3.1 Adolescent fertility rate

The model included significant terms for time, secondary but not primary education, GDP and for country income group (Table 2). There had been a 1.4% annual decrement in AFR across the study period, with each additional year of secondary education decreasing AFR by a further 8.4% independently of other factors. Primary education was not significant. Middle and high income countries had a lower baseline but a lesser decline over time than low income countries. Coefficients in the IPW model were larger, showing the controlled direct effect of each additional year of secondary education was a reduction in AFR by 14.6%. Each additional year of overall education decreased AFR by 9.4% (Table 3).

**Fig. 2:**
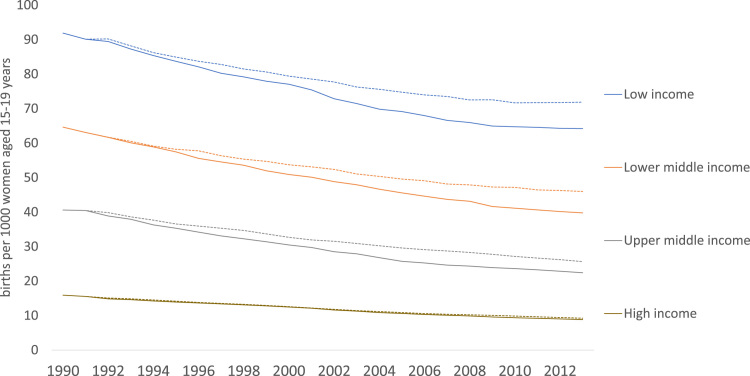
Counterfactual analysis of effect of secondary education progress on adolescent fertility by country income from 1990 to 2013.

Fig. 2 shows mean AFR across the study period in each of low, middle and high income countries, together with estimated fertility in the counterfactual scenario in which secondary education remained at the 1990 level but primary education and GDP increased as observed. Counterfactual analyses suggest that the proportion of the reduction in adolescent fertility rate over the study period independently attributable to secondary education was 28% in low income countries, 25% in lower middle, 18% in upper middle and 6% in high income countries. Counterfactual scenarios for the effect of secondary education on adolescent fertility in each region are shown in the Appendix (Fig A10), effects being greatest in the South Asia and Sub-Saharan Africa regions.

### 3.2 Mortality

Young women: In mixed effects models (Table 2), each additional year of secondary education reduced mortality by 4.5% for 15-19 year olds and 4.9% for 20-24 year olds. Coefficients from IPW models were larger, with each additional year of secondary education decreasing mortality by 16.9% for 15-19 year olds and 14.8% for 20-24 year olds. Models using WHO mortality data showed similar coefficients for secondary education (Appendix Table A2). The addition of HIV prevalence to mortality models resulted in no material change to education coefficients (Appendix Table A4).

**Fig. 3:**
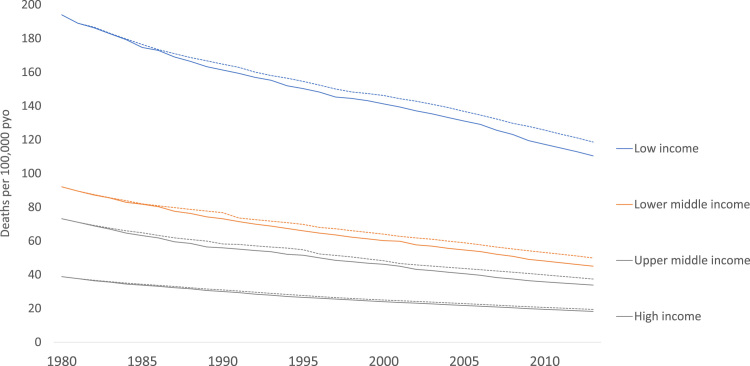
Counterfactual analysis of effect of secondary education progress on female mortality 15-19 years by country income from 1990 to 2013.

Estimated female mortality in the counterfactual scenario in which overall secondary education remained at the level it was in 1990 within each country is shown for 15-19 year olds in Fig. 3 and for 20-24 year olds in Appendix Figure A11. The proportion of the mortality reduction independently attributable to secondary education was 10% amongst 15-19 year olds and 19% and 14% amongst 20-24 year olds in low and middle income countries respectively, and 6% in high income countries in both age-groups. Counterfactual scenarios by region are shown in Appendix Figures A12 & 13, effects being greatest in the South Asia region.

Young men: Each additional year of secondary education reduced mortality by 2.5% for 15-19 year olds and 4.1% for 20-24 year olds (Table 2). Coefficients from structural marginal models were larger, with each additional year of secondary education decreasing mortality by 11.4% for 15-19 year olds and 8.8% for 20-24 year olds. Again, the addition of HIV prevalence to models (Appendix Table A4) and rerunning models using WHO mortality data (Appendix Table A2) resulted in no material change to coefficients.

Male mortality counterfactual analyses by country income and by region are shown in Appendix Figures A14-17. The proportion of the mortality reduction independently attributable to secondary education was 12% in low income, 7% in lower-middle, 3% in upper-middle and high income countries amongst 15-19 year olds and 23%, 10%, 6% and 4% respectively amongst 20-24 year olds.

### 3.3 HIV prevalence

**Fig. 4:**
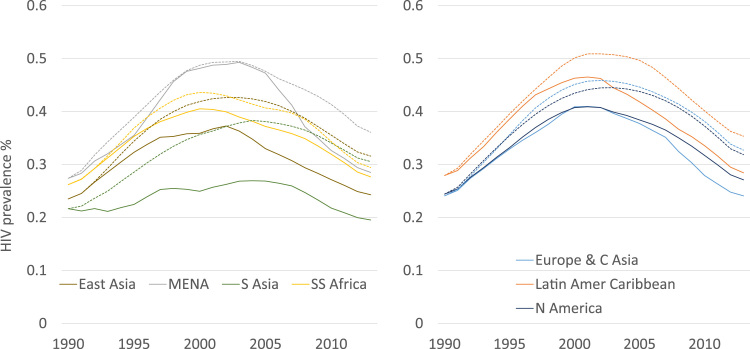
Counterfactual analysis of effect of secondary education progress on global HIV prevalence amongst males aged 15-24 years from 1990 to 2013 by region.

Models for HIV prevalence each included a significant quadratic term, consistent with the observed global rise and fall in HIV prevalence across the study period (Appendix Figure A20). Each additional year of secondary education was associated with a 24.5% reduction in HIV prevalence amongst males and 43.1% amongst females (Table 2). HIV prevalence estimates in the counterfactual scenario of no secondary education progress since 1990 are shown for young men by region in Fig. 4 (see Appendix Figures A18 & 19 for other estimates). Counterfactual analyses suggest that global HIV prevalence would have risen substantially further and remained higher across the study period in both sexes if secondary education in each country had remained at 1990 levels. The impact of education was particularly notable in South Asia, Latin America and Sub-Saharan Africa.

For each health outcome we reran mixed effects models additionally adjusting for national spending on health. Sample sizes were much reduced. Coefficients for secondary education for adolescent fertility and HIV prevalence were unchanged, however the significance of mortality coefficients was attenuated (Appendix Table A3).

## 4 Discussion

We found strong and consistent evidence that national progress in secondary education over the past three decades predicted major reductions in national adolescent fertility, all-cause mortality and HIV prevalence amongst young people, independently of growth in primary education, increasing national wealth and population growth. The health benefits associated with secondary education appeared greater than those associated with primary education and were greatest amongst young women. Amongst girls, each additional year of secondary education reduced adolescent fertility by 14.6%, mortality by up to 16.9% and HIV prevalence by 43%. Mortality and HIV reductions for young men related to secondary education were smaller but also highly significant. The benefits of secondary education for health were most marked amongst low income countries and particularly in South Asia and Sub-Saharan Africa.

This evidence was consistent across three priority health outcomes and two educational datasets studied here, and across a range of methods. Our findings complement existing individual-level evidence for education as a potential tool to improve population health, (Baker et al., 2011, Behrman, 2015, Masters et al., 2015, Miyamoto and Chevalier, 2010, Montez and Friedman, 2015) and show that this association appeared to be consistent across a range of country income and levels of national development. Our findings suggest that country-level investments in secondary education are likely to result in major health dividends for the most productive parts of the population and the parents of the next generation.

Mean years of secondary education increased by approximately 60% globally between 1980 and 2010, with the greatest growth in upper middle income countries who have therefore reaped the greatest health benefits. Target 4.1 of the SDGs is that all young people complete secondary education by 2030, (Sustainable Development Knowledge Platform, 2014) driven largely by economic benefit to countries (Barro, 2013). Investment in secondary education will also bring a major health benefit for low and lower-middle income countries who meet this target.

### 4.1 Comparison with literature

Ours is the first systematic study of the health benefits associated with secondary education globally, although it builds upon and informs previous work on education and population (Lutz & Kc, 2011) and on childhood mortality (Gakidou et al., 2010).

A large literature has examined the relationships between length of education and teenage child-bearing, concluding that despite endogeneity between health and education, higher levels of education are protective against sexual debut and teenage child bearing across high and low income settings (Alsan & Cutler, 2013; Duflo, Dupas, & Kremer, 2015; McQueston, Silverman, & Glassman, 2013). However, few have specifically examined secondary education. Our findings were consistent with those of Mahy & Gupta (2002), who reported that the protective effect of secondary schooling on teenage fertility was stronger than that of primary education across 8 Sub Saharan African countries (compared with no schooling, odds ratios (OR) for childbearing <18 years related to ≥8 years of schooling were 0.09 to 0.47 and significant in all countries, whilst OR for primary (1-7 years) schooling were largely non-significant) (Mahy & Gupta, 2002).

Given that both HIV and teenage child-bearing share some common antecedents in terms of sexual behaviours, education may have similar effects on both. Education is suggested to be protective against HIV through mechanisms including improved decision-making leading to a range of protective behaviour changes, (Jukes et al., 2008) better general health and reduced exposure to older partners (Hargreaves et al., 2008). Our findings are consistent with previous studies which suggest that secondary education is most protective, (de Walque et al., 2005, Gregson et al., 2001) with the size of the effect identified for each additional year of schooling similar to previous studies (de Walque et al., 2005). Our finding that secondary education had significant effects on HIV prevalence whilst overall education did not is consistent with the non-significant positive coefficients for primary education in our models and suggests a U-shaped relationship of education with HIV prevalence.

We are aware of no previous international studies of associations of youth mortality with secondary education. However our findings are consistent with a large literature showing education reduces adult mortality independent of socioeconomic status (Baker et al., 2011). In adult women in Zimbabwe, each additional year of secondary education was estimated to be associated with a 21% decline in mortality amongst their children, (Grepin & Bharadwaj, 2015) similar to the 17% benefit we identified for young women. Our findings relate to all-cause mortality, although it is likely that there are differing associations with different causes of mortality that vary by sex and by national development.

Others have suggested that the relationship of education and health depend on national development or stage of the epidemiological transition (Smith, Anderson, Salinas, Horvatek, & Baker, 2015). Whilst our ecological level analyses cannot directly contribute to identifying the socioeconomic contexts in which education and health may be most strongly or causally related, our models suggested that the greatest benefits of expanded education for health would be in sub-Saharan Africa, South Asia and Latin America, although benefits are seen across all regions. The former relationship is consistent with resource substitution theories, which suggest that the benefits of education for health are greatest in those with limited resources for establishing and maintaining good health (Ross & Mirowsky, 2011). Others have previously noted that Sub-Saharan Africa is the global region with the lowest gains in secondary schooling yet persistently high fertility, adolescent fertility and child mortality (Mingat, Ledoux, & Rakotomalal, 2010). Whilst there are undoubted challenges in financing expansion in secondary education in regions such as sub-Saharan Africa, (Mingat et al., 2010) our data suggest that investment in secondary education to meet Target 4.1 of the SDGs will bring a significant health benefit to countries in addition to the economic dividends.

We found little evidence for health gains from expansion of secondary education in high income countries, potentially reflecting already high levels of secondary schooling, different associations between secondary education and health in such settings or the dilution of secondary education effects by progress through higher education. However it is possible that identified benefits for health from tertiary education (Case & Deaton, 2015) may be predicated on successful completion of secondary education.

### 4.2 Strengths and limitations

We used recently available time-series data on educational participation from two sources to study relationships of secondary education and health outcomes. As primary and secondary education are necessarily related, with secondary education only possible after largely completing primary, we used two methods to estimate the independent effects of secondary education on outcomes. First, we included both primary and secondary education as time-varying predictors in multilevel models, each centred to reduce issues of collinearity. Secondly we used structural marginal models in which inverse probability weights allowed us to estimate the direct effects of secondary education independently of primary education, regardless of potential collinearity between the two. We considered modelling education as a categorical predictor, however we felt this was a less satisfactory solution for a number of reasons. First, converting continuous measures to categorical predictors reduces statistical power, particularly if multiple categories are used. Second, many individuals in LMIC only complete part of primary or secondary education, thus simple categories of primary or secondary education are likely to result in misclassification bias. Third, the requirement for a referent category for analyses would not allow us to examine the contribution of secondary education adjusted for primary education, but only in comparison with primary.

Our analyses for secondary education took account of time-varying confounding by primary education and GDP. In additional analyses we took account of changing healthcare spending. Coefficients were larger in IPW models than in standard mixed effects models, suggesting that time varying confounding by GDP or primary education was accounted for in IPW models. Neither the BL nor IHME education datasets used any of the outcomes or covariates (e.g. GDP) studied here to model missing data. Whilst missing data in the IHME mortality dataset were modelled using educational data, repeating analyses using unmodelled WHO mortality data showed coefficients for secondary education of similar magnitude.

Country-level analyses of data collected at the individual level may produce confounded findings, the ‘ecological fallacy’. Further, our analyses provide no information on mechanisms by which expanded education may influence health. However we believe there are strong reasons to conclude the associations shown here are sufficient for countries and international agencies to act upon. First, in each of HIV, mortality and adolescent fertility, detailed studies using randomized trials, (Brent, 2009) natural experiments (Grepin & Bharadwaj, 2015) or sophisticated causal analysis techniques (Ferre, 2009) have concluded that there are direct benefits of increased education for health. Second, we studied health outcomes at country level and limit our conclusions to the country level. Third, our analyses were longitudinal and took into account two measures of income (country GDP and country income group), the most likely mediators of relationships between education and health, as well as country-level health spending. Health spending made no material changes to models for HIV or adolescent fertility although it attenuated the significance of mortality coefficients, likely due to the much reduced sample size.

### 4.3 Conclusions

Investment by countries in broadening access to secondary education promises health benefits of a similar or greater order than those seen with primary education. Secondary education has the potential to be a social vaccine with broad efficacy for multiple health outcomes in low and middle income countries. Meeting the SDG target of universal secondary education will be a key mechanism for achieving the health targets in SDG 3. Work to achieve universal education by the International Commission on Financing Global Education and others need to look beyond economic benefits to include health gains from secondary education.

## Funding

This research did not receive any specific grant from funding agencies in the public, commercial, or not-for-profit sectors.
