# Supplementary material for: The health benefits of secondary education in adolescents and young adults: An international analysis in 186 low-, middle- and high-income countries from 1990 to 2013
Source: SSM Popul Health. 2016 Dec 14;3:162–71. doi: 10.1016/j.ssmph.2016.12.004 (PMC5742637; doi:10.1016/j.ssmph.2016.12.004)
Supplement: Supplementary file 1 — Supplementary material [file mmc1.docx]

# Appendices

## Appendix A. METHODS

Additional information on methods to those presented in the main paper are given here.

### Education data

1. Barro Lee dataset.

Barro Lee (BL) data were downloaded from http://www.barrolee.com/data/wholepop.htm on 18 November 2015. Details of the derivation of the BL data on average completed years of education can be found on the BL website (<http://www.barrolee.com)> and in a National Bureau of Economic Research working paper.(Barro & Lee, 2010) Data were compiled from country-level census data and surveys, with missing observations completed by forward and backward extrapolation of the census/survey observations by age group. Data on the distribution of educational attainment among the population, combined with the information for each country on the duration of school at each level, were used to generate the number of years of schooling achieved by the average person at various levels of education (i.e. primary, secondary, tertiary).(Barro & Lee, 2010)

Here we used estimates for 15-19 and 20-24 year old males and females for each year 1980 through to 2010, with data for 20-24 year olds used in our primary exposure variable for analyses of outcomes in both 15-19 and 20-24 year olds. This was done as we considered that many 15-19 year olds would not have completed secondary education despite opportunities and access to do so in a country, whereas 20-24 year olds would (almost) all have completed whatever secondary education opportunities were available in a country and were sufficiently close in age/period to 15-19 year olds to also provide an educational proxy for that age group.

Data were available in 5 year intervals for each decade and half decade. To improve model fit and power, given that outcome data were available for individual years, we used a smoothing algorithm in Stata to estimate BL secondary and primary education data for each year 1980 to 2013. For year 1 of each half decade we used the preceding BL data point. For Years 2 & 3 we averaged the preceding (i.e. Year 0) and next (Year 5) half-decade data point. For Year 4 we used the next (Year 5) data point.

For longitudinal models, secondary and primary education were centred on 1980 or 1990 as appropriate.

2. Institute of Health Evaluation and Metrics (IHME) dataset

IHME education data on average years of overall education per country were downloaded on 29 April 2015 from <http://ghdx.healthdata.org/record/global-educational-attainment-1970-2015>. Further details regarding the data can be found in the IHME report A Hand Up: Global Progress Towards Universal Education (2010).(Institute for Health Metrics and Evaluation (IHME), 2015) Estimates were obtained from all available country-level census data and nationally representative surveys gathered between 1953 and 2015. For surveys or censuses that included questions about the number of years of completed education, these data were used directly. Where surveys asked respondents about the highest form of education they had attended and the highest grade they had completed at that level, years of schooling was calculated using country-specific information about school cycles from UNESCO’s database of national education systems. Data sources in which the education variables were missing for more than 10% of respondents were not used. When individual level data were not accessible, tabulated information presented in reports or UN demographic yearbooks was used. Estimates for country years without actual data were made available data using mixed effects models, which were then combined with all available data by use of Loess regression to produce a time series of educational data.(Gakidou et al., 2010; Institute for Health Metrics and Evaluation (IHME), 2015)

Data were available in 10 year age-bands and for these analyses we used data on 15-24 year olds, recognizing that some in this age-band would not have completed all secondary education opportunities available to them in a country

### Mortality data

1. Global Burden of Disease 2013 study data

Global Burden of Disease 2013 study mortality data from the IHME were downloaded on 29 July 2015 from https://cloud.ihme.washington.edu/index.php/s/b89390325f728bbd99de0356d3be6900. We restricted these analyses to total / all-cause mortality. Data were available as total deaths and mortality rate per 100,000 population by 5 year age group, and here we used mortality rate data on 15-19 and 20-24 year olds. Data were available for 187 countries for each year from 1980 to 2013. Countries ranged across the spectrum from low income through to high income countries. There were high levels of missing data for low and lower middle income countries. IHME publications show that for the development of the complete data series for each country, all available vital registration data, sibling history survey data, sample registration data, and household recall of deaths were systematically identified for that country. Missing data were synthesized using a combination of spatiotemporal regression and Gaussian process regression, the latter including income per person, mean years of education in age group 15–60 years, mortality caused by HIV/AIDS, and country random effects as covariates.(Institute for Health Metrics and Evaluation (IHME), 2014; Mortality & Causes of Death, 2015)

2. WHO World Mortality Database

Data were downloaded from the World Mortality Database (<http://www.who.int/healthinfo/statistics/mortality_rawdata/en/)> on 6 December 2015. The WMD base comprises deaths and cause of death (ICD 7 to 10) registered by WHO Member States from 1950. Data were obtained from 1980 onwards, aggregated within the age groups 15-19 and 20-24 years and stratified by sex. We calculated death rates per 100,000 population or 100,000 person years of observation (pyo) in the specific age-group using WMD population data, or where missing, from UN population estimates. Few countries had contiguous data from 1980 onwards and data varied in completeness between years within countries. WHO assessments of levels of completeness of death registration data were used to reject unreliable data (see <http://www.who.int/healthinfo/morttables/en/)>. Whilst data were available for 87 countries for secondary education analyses, these constituted only a total of 429 country-years – with a few high income countries contributing full series of country-years (i.e. 33 years from 1980 to 2013) and the few low and middle income countries included contributing very incomplete data series.

### Adolescent fertility rate

Data were obtained from the United Nations, Department of Economic and Social Affairs, Population Division 2014 *Update for the MDG Database*, downloaded on 29 May 2015 from [www.unpopulation.org](http://www.unpopulation.org). Data were obtained from a number of sources including national vital registration systems and a range of national and international surveys.(United Nations, 2014) Data were available for 225 countries from 1990 to 2012, with a variable number of years available per country ranging from 1 to 22.

HIV prevalence

### Macroeconomic data

Macroeconomic data were obtained in November 2015 using the *wbopendata* command in Stata, a command written by the World Bank which retrieves data directly from the World Bank Open Data dataase (see <http://data.worldbank.org/news/accessing-world-bank-open-data-in-stata)>.

Gross domestic product (GDP): GDP data were downloaded as national GDP per capita in international $US using purchasing price parity.

Country income status was obtained by categorizing countries as high, upper-middle, lower-middle or low income countries according to historical World Bank data on country income status derived from gross national income, downloaded from <https://datahelpdesk.worldbank.org/knowledgebase/articles/378834-how-does-the-world-bank-classify-countries> . For these analyses, we used classifications for 1990, being the baseline for most of our analyses.

Data on total health spending as a proportion of national GDP for each country were obtained as indicator SH.XPD.TOTL.ZS. This refers to total national health expenditure, which is the sum of public and private health expenditure, covering the provision of health services (preventive and curative), family planning activities, nutrition activities, and emergency aid designated for health but does not include provision of water and sanitation. Data were available from 1995 to 2013 for 188 countries. Data source: World Health Organization Global Health Expenditure database.

### Analyses

Detail is provided here to amplify the descriptions given in the main paper.

Cross-sectional models: Models were run using data from 2010, being the last data point of the BL education dataset and also the year for which maximum health outcome data were available. For outcomes, where data were not available for 2010, the closest available data from after 2001 were used; if the latest available data were for a year before 2001, that country was counted as missing in these analyses. Models were run using linear regression to assess the relationship of i) secondary and primary education and of ii) overall education with each of the health outcomes by age-group and gender. Outcomes were modelled as natural logarithms as each was notably skewed.

Mixed effect models: These included random effects for time and country and were of the following form, where c=country and t=time in years:

Y_c,t_ = Intercept + β₀Time+β₁Secondary education_c,t_ + β_2_Primary education_c,t_ + β_3_GDP_c,t_+ β_4_(country income status) + β_5_(interaction country income status by time) + αc + αt + ε_c,t_

Country income status was added as a fixed effect to ascertain the impact of country income status on baseline intercept, with an interaction term between income status and year included to model the effect of country status on slope. Country income status and its interaction with time were significant in and improved model fit of most models, supporting our contention that this variable added country level context over and above time varying GDP measures.

Time and educational data (secondary, primary and overall education) were centred on the initial year (1980 for mortality, 1990 for teenage fertility and HIV) to minimise collinearity. Models were tested for quadratic terms for time and education and for interactions between education and time, with interactions included if p<0.1. Quadratic terms for time were only significant for HIV models, consistent with the rise and fall in HIV prevalence over the study period. There were no significant quadratic terms for education identified.

IPW models: We constructed stabilized IP weights following accepted methods(Hernan & Robins, forthcoming) and regressing the exposure (secondary education) onto time varying potential confounders, log of 10 year lagged GDP and log of country population in each year, plus country income status. Weights were calculated separately by sex. We followed Fewell et al. 2004 in assuming the intercept for time is a smooth function and to estimate this using cubic splines. This allowed us to then estimate a pooled weighted regression equation, where each country’s level of education at each time point is unrelated to its time-updated covariates.(Fewell et al., 2004) For the analyses for overall education, inverse probability weights were constructed to include time-varying variables (log of lagged GDP and log of country population of 15-24 year olds in each year) and country income status. IPW models were not estimated for HIV due to inclusion of a significant quadratic term in the models

Counterfactuals: Counterfactual scenarios were estimated from coefficients in the mixed effects models using the *predict* command in Stata. The counterfactual scenario assessed here was of no progress in secondary education in countries after 1980 (for mortality) or 1990 (for adolescent fertility and HIV prevalence). That is, the counterfactual estimated change in the outcome from 1980/1990 to 2013 if secondary education in each country had stayed at the level it was in 1980/1990. Counterfactuals were estimated by running the regression equation with actual secondary education data then replacing secondary education data in each country for all years after 1980/1990 with average years of secondary education in that country in 1980/1990, and then rerunning the *predict* command to estimate the counterfactual. This was repeated by country income status and by UN region.

## APPENDIX B. ADDITIONAL FIGURES & TABLES

### Figure A1. Educational progress amongst males 15-24 years from 1980 to 2013 by country income

LIC low income country; LMIC lower middle income country; UMIC upper middle income country; HIC high income country

Graph shows median (solid line) and 25^th^ and 75% centiles (lower & upper dotted lines) for each income group (coloured by income group).

### Figure A2. Educational progress amongst females 15-24 years from 1980 to 2013 by country income

LIC low income country; LMIC lower middle income country; UMIC upper middle income country; HIC high income country

Graph shows median (solid line) and 25^th^ and 75% centiles (lower & upper dotted lines) for each income group (coloured by income group).

### Figure A3. Secondary education and female mortality 15-24 years in 2010

Panel A

Panel B.

### Figure A4. Secondary education and male mortality 20-24 years in 2020

### Fig A5. HIV prevalence and years of secondary education in males in 2010

### Figure A6. Overall education and adolescent fertility rate in 2010

### Fig. A7. Overall education and male mortality 15-24 years in 2010

Panel A

Panel B

### Fig A8. Overall education and female mortality 15-24 years in 2010

Panel A

Panel B

### Fig A9. Overall education and HIV prevalence amongst males and females 15-24 years

Males

Females

### Table A1. Cross-sectional associations between secondary years of education per capita and mortality amongst 15-24 year olds (from WHO World Mortality Database) in 2010 by sex

| Outcome |  | N | Coefficient | Exponentiated coefficient | p |
| --- | --- | --- | --- | --- | --- |
| **Mortality rate** |  | 72 countries |  |  |  |
| (log deaths/100,000) |  |  |  |  |  |
| 15-19 year males | Primary years |  | -0.067 | 0.935 | 0.2 |
|  | Secondary years |  | -0.077 | 0.926 | 0.07 |
| 15-19 year females | Primary years |  | -0.114 | 0.892 | <0.0001 |
|  | Secondary years |  | -0.065 | 0.937 | <0.0001 |
| 20-24 year males | Primary years |  | -0.076 | 0.926 | <0.0001 |
|  | Secondary years |  | -0.020 | 0.980 | 0.03 |
| 20-24 year females | Primary years |  | -0.067 | 0.935 | 0.2 |
|  | Secondary years |  | -0.103 | 0.902 | 0.01 |
|  |  |  |  |  |  |
| Note: All models adjusted for log of mean GDP for previous decade (2001-2010) and income status for each country. Secondary and primary years of education were entered together in the same model | | | | | |

### Table A2. Mixed effect models for mortality using the WHO World Mortality Database: Models for effects of secondary and primary education on mortality for males and females, 1995 to 2013

|  | **Male mortality** | | | | | | | **Female mortality** | | | | | |
| --- | --- | --- | --- | --- | --- | --- | --- | --- | --- | --- | --- | --- | --- |
|  | 429 country-years | | | | | | | 429 country-years | | | | | |
|  | across 82 countries | | | | | | | across 82 countries | | | | | |
|  | (log deaths per 100,000 pyo) | | | | | | | (log deaths per 100,000 pyo) | | | | | |
|  |  | 15-19 year males | | | 20-24 year males | | | 15-19 year females | | | 20-24 year females | | |
| **Mixed effect models** |  | *B* | *ExpB** | *p* | *B* | *ExpB** | *p* | *B* | *ExpB** | *p* | *B* | *ExpB** | *p* |
| Constant |  | 6.071 |  |  | 5.145 |  |  | 4.107 |  |  | 4.325 |  |  |
| Time (years) | linear | -0.003 | 0.997 | 0.6 | -0.009 | 0.991 | 0.001 | -0.013 | 0.987 | <0.001 | -0.012 | 0.988 | <0.001 |
| *Education* |  |  |  |  |  |  |  |  |  |  |  |  |  |
| Primary education per capita (years) | | 0.019 | 1.019 | 0.7 | -0.070 | 0.933 | 0.02 | -0.049 | 0.952 | 0.04 | -0.065 | 0.937 | 0.06 |
| Secondary education per capita (years) | | -0.082 | 0.921 | 0.04 | -0.057 | 0.944 | 0.04 | -0.043 | 0.958 | 0.02 | -0.050 | 0.951 | 0.01 |
| GDP (log $ per annum) |  | -0.176 | 0.839 | 0.03 | - |  |  | - |  |  | - |  |  |
|  |  |  |  |  |  |  |  |  |  |  |  |  |  |

Note:

Country income was not significant in these models as very few low and lower middle income countries

GDP not included in models where not significant to improve model fit

Pyo: person years of observation

B: coefficient

ExpB: exponentiated coefficient

### Table A3. Mixed effect models including health spending as proportion of GDP: Models for effects of secondary and primary education on adolescent fertility, mortality and HIV prevalence for males and females, 1995 to 2013

|  |  | | **Adolescent fertility rate** | | | **Male mortality** | | | | | | **Female mortality** | | | | | | HIV prevalence | | | | | |
| --- | --- | --- | --- | --- | --- | --- | --- | --- | --- | --- | --- | --- | --- | --- | --- | --- | --- | --- | --- | --- | --- | --- | --- |
|  |  | | 1330 country-years across 136 countries | | | 2594 country-years across 139 countries | | | | | | 2594 country-years across 139 countries | | | | | | 1681 country-years across 91 countries | | | | | |
|  |  | | (log births per 1000 women 15-19 years) | | | (log deaths per 100,000 pyo) | | | | | | (log deaths per 100,000 pyo) | | | | | | % population aged 15-24 years | | | | | |
|  |  | |  |  |  | 15-19 year males | | | 20-24 year males | | | 15-19 year females | | | 20-24 year females | | | 15-24 year males | | | 15-24 year females | | |
| **Mixed effect models** |  | | *B* | *ExpB** | *p* | *B* | *ExpB** | *p* | *B* | *ExpB** | *p* | *B* | *ExpB** | *p* | *B* | *ExpB** | *p* | *B* | *ExpB** | *p* | *B* | *ExpB** | *p* |
| Constant |  | | 3.066 |  |  | 5.615 |  |  | 6.206 |  |  | 5.956 |  |  | 6.941 |  |  | -2.317 |  |  | -1.430 |  |  |
| Time (years) | linear | | -0.009 | 0.991 | 0.18 | 0.000 | 1.000 | 0.9 | 0.000 | 1.000 | 0.9 | -0.016 | 0.984 | <0.0001 | -0.010 | 0.990 | 0.06 | 0.036 | 1.036 | .10 | 0.006 | 1.006 | 0.4 |
|  | quadratic | |  |  |  |  |  |  |  |  |  |  |  |  |  |  |  | -0.002 | 0.998 | 0.02 | -0.002 | 0.998 | 0.003 |
| *Education* |  | |  |  |  |  |  |  |  |  |  |  |  |  |  |  |  |  |  |  |  |  |  |
| Primary education per capita (years) | | 0.029 | | 1.029 | 0.3 | 0.013 | 1.013 | 0.2 | 0.005 | 1.005 | 0.9 | 0.003 | 1.003 | 0.9 | 0.001 | 1.001 | 0.9 | -0.022 | 0.978 | 0.8 | -0.063 | 0.939 | 0.5 |
| Secondary education per capita (years) | | -0.093 | | 0.911 | 0.002 | -0.009 | 0.991 | 0.4 | -0.019 | 0.981 | 0.4 | -0.020 | 0.980 | 0.4 | -0.019 | 0.981 | 0.5 | -0.330 | 0.719 | <0.001 | -0.318 | 0.728 | 0.001 |
| GDP (log $ per annum) | | 0.226 | | 1.253 | 0.002 | -0.052 | 0.949 | 0.04 | -0.060 | 0.942 | 0.03 | -0.105 | 0.900 | <0.001 | -0.193 | 0.824 | <0.001 | 0.163 | 1.177 | 0.3 | 0.002 | 1.002 | 0.9 |
| Country status | Low income | 0 | |  |  | 0 |  |  | 0 |  |  | 0 |  |  | 0 |  |  | - |  |  | - |  |  |
|  | Lower middle | -0.580 | | 0.560 | 0.006 | -0.285 | 0.752 | 0.006 | -0.419 | 0.658 | 0.001 | -0.805 | 0.447 | 0.15 | -0.858 | 0.424 | <0.001 | - |  |  | - |  |  |
|  | Upper middle | -1.147 | | 0.318 | <0.001 | -0.127 | 0.880 | 0.4 | -0.335 | 0.715 | 0.07 | -0.778 | 0.459 | 0.6 | -0.813 | 0.443 | <0.001 | - |  |  | - |  |  |
|  | High income | -2.228 | | 0.108 | <0.001 | -0.390 | 0.677 | 0.008 | -0.396 | 0.673 | 0.02 | -1.067 | 0.344 | <0.001 | -1.065 | 0.345 | <0.001 | - |  |  | - |  |  |
| Health spending (% of GDP) | | 0.007 | | 1.007 | 0.4 | -0.001 | 0.999 | 0.8 | -0.003 | 0.997 | 0.2 | -0.005 | 0.995 | 0.15 | -0.007 | 0.993 | 0.15 | 0.012 | 1.012 | 0.4 | 0.000 | 1.000 | 0.9 |
| Interaction of income status with time | | -0.005 | |  | 0.04 | -0.007 |  | <0.001 | -0.006 |  | <0.001 | -0.001 |  | 0.6 | -0.003 |  | 0.06 | - |  |  | - |  |  |

Table Notes:

Pyo: person years of observation

B: coefficient

ExpB: exponentiated coefficient

### Fig A10. Counterfactual analysis of effect of secondary education progress on adolescent fertility by region from 1990 to 2013

Dotted lines of each colour show counterfactual estimates for each region for the scenario in which secondary education remained at 1990 levels in each country

### Table A4. Global models for mortality including HIV prevalence: Table shows associations of (a) secondary and primary and (b) overall education on mortality amongst males and females, 1980 to 2013

|  | | | | Male mortality | | | | Female mortality | | | | | |
| --- | --- | --- | --- | --- | --- | --- | --- | --- | --- | --- | --- | --- | --- |
|  |  | 2158 country-years    across 93 countries | | | | | | 2158 country-years    across 93 countries | | | | | |
| **Models for secondary and primary education (BL)** | |  |  |  |  |  |  |  |  |  |  |  |  |
|  |  | 15-19 year males | | | 20-24 year males | | | 15-19 year females | | | 20-24 year females | | |
|  |  | *Coefficient* | *Exp* coefficient* | *p* | *Coefficient* | *Exp* coefficient* | *p* | *Coefficient* | *Exp* coefficient* | *p* | *Coefficient* | *Exp* coefficient* | *p* |
| Constant |  | 5.554 |  |  | 6.049 |  |  | 5.713 |  |  | 6.896 |  |  |
| Time (years) | linear | -0.010 | 0.990 | <0.0001 | -0.008 | 0.992 | <0.0001 | -0.016 | 0.984 | <0.0001 | -0.013 | 0.987 | <0.0001 |
|  | quadratic |  |  | 0.05 |  |  |  |  |  |  |  |  |  |
| *Education* |  |  |  |  |  |  |  |  |  |  |  |  |  |
| Primary education per capita (years) | | -0.018 | 0.983 | 0.05 | -0.002 | 0.998 | 0.9 | -0.013 | 0.987 | 0.4 | 0.009 | 1.009 | 0.8 |
| Secondary education per capita (years) | | -0.023 | 0.977 | 0.04 | -0.031 | 0.969 | 0.09 | -0.059 | 0.943 | 0.06 | 0.042 | 1.043 | 0.5 |
| GDP (log $ per annum) |  | -0.040 | 0.961 | 0.03 | -0.046 | 0.955 | 0.04 | -0.071 | 0.932 | 0.001 | -0.140 | 0.870 | <0.0001 |
| HIV prevalence (log %) |  | 0.049 | 1.051 | <0.0001 | 0.093 | 1.098 | <0.0001 | 0.113 | 1.119 | <0.0001 | 0.257 | 1.293 | <0.0001 |
| Country income status | Low income | Reference |  |  | Reference |  |  | Reference |  |  | Reference |  |  |
|  | Lower middle | -0.186 | 0.830 | 0.08 | -0.173 | 0.841 | 0.12 | -0.566 | 0.568 | <0.0001 | -0.771 | 0.463 | 0.002 |
|  | Upper middle | -0.061 | 0.941 | 0.7 | 0.116 | 1.123 | 0.6 | -0.573 | 0.564 | 0.01 | -1.073 | 0.342 | <0.0001 |
|  | High income | -0.429 | 0.651 | 0.01 | -0.484 | 0.616 | 0.002 | -1.129 | 0.323 | <0.0001 | -1.269 | 0.281 | <0.0001 |
|  |  |  |  |  |  |  |  |  |  |  |  |  |  |
|  |  | 2606 country-years    across 113 countries | | | | | | 2606 country-years    across 113 countries | | | | | |
| **Models for overall education (IHME)** | |  |  |  |  |  |  |  |  |  |  |  |  |
|  |  | 15-19 year males | | | 20-24 year males | | | 15-19 year females | | | 20-24 year females | | |
|  |  | *Coefficient* | *Exp* coefficient* | *p* | *Coefficient* | *Exp* coefficient* | *p* | *Coefficient* | *Exp* coefficient* | *p* | *Coefficient* | *Exp* coefficient* | *p* |
| Constant |  | 5.125 |  |  | 5.639 |  |  | 5.070 |  |  | 5.546 |  |  |
| Time (years) | linear | 0.001 | 1.001 | 0.8 | 0.001 | 1.001 | 0.7 | -0.012 | 0.988 | 0.01 | -0.003 | 0.997 | 0.5 |
| Education per capita (years) | | -0.101 | 0.904 | <0.0001 | -0.087 | 0.916 | 0.01 | -0.050 | 0.952 | 0.07 | -0.080 | 0.923 | 0.01 |
| GDP (log $ per annum) |  | -0.026 | 0.975 | 0.10 | -0.035 | 0.965 | 0.06 | -0.049 | 0.952 | 0.3 | -0.113 | 0.893 | 0.05 |
| HIV prevalence (log %) |  | 0.041 | 1.042 | <0.0001 | 0.085 | 1.089 | <0.0001 | 0.132 | 1.142 | <0.0001 | 0.264 | 1.303 | <0.0001 |
| Country income status | Low income | Reference |  |  | Reference |  |  | Reference |  |  | Reference |  |  |
|  | Lower middle | 0.006 | 1.006 | 0.9 | 0.010 | 1.010 | 0.9 | -0.453 | 0.636 | <0.0001 | -0.282 | 0.754 | 0.04 |
|  | Upper middle | 0.164 | 1.179 | 0.13 | 0.198 | 1.219 | 0.2 | -0.588 | 0.555 | <0.0001 | -0.434 | 0.648 | 0.03 |
|  | High income | -0.129 | 0.879 | 0.4 | -0.295 | 0.745 | 0.13 | -1.043 | 0.352 | <0.0001 | -0.734 | 0.480 | 0.007 |

### Figure A11. Counterfactual analysis of effect of secondary education progress on female mortality 20-24 years by country income from 1980 to 2013

Dotted lines of each colour show counterfactual estimates for each income group for the scenario in which secondary education remained at 1980 levels in each country

### Figure A12. Counterfactual analysis of effect of secondary education progress on female mortality 15-19 years by region from 1980 to 2013

Dotted lines of each colour show counterfactual estimates for each region for the scenario in which secondary education remained at 1980 levels in each country

### Figure A13. Counterfactual analysis of effect of secondary education progress on female mortality 20-24 years by region from 1980 to 2013

Dotted lines of each colour show counterfactual estimates for each region for the scenario in which secondary education remained at 1980 levels in each country

### Figure A14. Counterfactual analysis of effect of secondary education progress on male mortality 15-19 years by country income from 1980 to 2013

Dotted lines of each colour show counterfactual estimates for each income group for the scenario in which secondary education remained at 1980 levels in each country

### Figure A15. Counterfactual analysis of effect of secondary education progress on male mortality 15-19 years by region from 1980 to 2013

Dotted lines of each colour show counterfactual estimates for each region for the scenario in which secondary education remained at 1980 levels in each country

### Figure A16. Counterfactual analysis of effect of secondary education progress on 20-24 year male mortality by country income from 1980 to 2013

Dotted lines of each colour show counterfactual estimates for each income group for the scenario in which secondary education remained at 1980 levels in each country

### Figure A17. Counterfactual analysis of effect of secondary education progress on male mortality 20-24 years by region from 1980 to 2013

Dotted lines of each colour show counterfactual estimates for each region for the scenario in which secondary education remained at 1980 levels in each country

### Figure A18. Counterfactual analysis of effect of secondary education progress on global HIV prevalence amongst females aged 15-24 years from 1990 to 2013 by region

Dotted lines of each colour show counterfactual estimates for each region for the scenario in which secondary education remained at 1990 levels in each country

### Figure A19. Counterfactual analysis of effect of secondary education progress on global HIV prevalence amongst males aged 15-24 years from 1990 to 2013 by country income

Dotted lines of each colour show counterfactual estimates for each income group for the scenario in which secondary education remained at 1990 levels in each country

### Figure A20. Median observed global HIV prevalence for males and females aged 15-24 years from 1990 to 2013

Note: Figure shows 3 year moving averages of global median prevalence

### References

Barro, R.J., & Lee, J.-W. (2010). A new dataset of educational attainment in the world 1950-2010. Cambridge, MA: National Bureau of Economic Research.

Fewell, Z., Wolfe, F., Choi, H., Hernan, M.A., Tilling, K., & Sterne, J.A.C. (2004). Controlling for time-dependent confounding using marginal structural models. *The Stata Journal,* 4, 402-420.

Gakidou, E., Cowling, K., Lozano, R., & Murray, C.J. (2010). Increased educational attainment and its effect on child mortality in 175 countries between 1970 and 2009: a systematic analysis. *Lancet,* 376, 959-974.

Hernan, M.A., & Robins, J.M. (forthcoming). Inverse probability weighting and structural marginal models. Causal inference pp. 11-22). Boca Raton: Chapman & Hall/CRC.

Institute for Health Metrics and Evaluation (IHME). (2014). Global Burden of Disease Study 2013. Global Burden of Disease Study 2013 (GBD 2013) Age-Sex Specific All-Cause and Cause-Specific Mortality 1990-2013. Seattle, United States: Institute for Health Metrics and Evaluation (IHME).

Institute for Health Metrics and Evaluation (IHME). (2015). Global Educational Attainment 1970-2015. Seattle, USA: Institute for Health Metrics and Evaluation (IHME).

Mortality, G.B.D., & Causes of Death, C. (2015). Global, regional, and national age-sex specific all-cause and cause-specific mortality for 240 causes of death, 1990-2013: a systematic analysis for the Global Burden of Disease Study 2013. *Lancet,* 385, 117-171.

United Nations, D.o.E.a.S.A., Population Division, . (2014). 2014 Update for the MDG Database:vAdolescent Birth Rate (POP/DB/Fert/A/MDG2014). . United Nations Population Division.
